# Diagnostic Value of Serum sST2 and MicroRNA-29a in Ovarian Cancer: A Dual-Biomarker Pilot Study

**DOI:** 10.3390/cimb48010113

**Published:** 2026-01-21

**Authors:** Fatma Tuba Akdeniz, Zerrin Barut, Orcun Avsar, Selvi Duman Bakırezer, Rukset Attar, Turgay Isbir

**Affiliations:** 1 Department of Genetics and Bioengineering, Faculty of Engineering and Natural Sciences, Istanbul Okan University, Istanbul 34959, Türkiye; 2 Department of Basic Medical Sciences, Faculty of Dentistry, Antalya Bilim University, Antalya 07190, Türkiye; zerrin.barut@antalya.edu.tr; 3 Department of Molecular Biology & Genetics, Faculty of Engineering and Natural Sciences, Hitit University, Corum 19030, Türkiye; orcunavsar@hitit.edu.tr; 4 Department of Basic Medical Sciences, Faculty of Medicine, Yeditepe University, Istanbul 34755, Türkiye; selvi.duman@yeditepe.edu.tr; 5 Department of Obstetrics and Gynecology, School of Medicine, Yeditepe University, Istanbul 34755, Türkiye; rattar@yeditepe.edu.tr; 6 Department of Molecular Medicine, Institute of Health Science, Yeditepe University, Istanbul 34755, Türkiye

**Keywords:** ovarian cancer, microRNA-29a, sST2, receiver operating characteristic analysis, biomarker, qPCR, ELISA

## Abstract

Ovarian cancer is frequently diagnosed at an advanced stage due to non-specific symptoms, contributing to high mortality. The limited diagnostic performance of current serum assays in early disease underscores the need for complementary circulating biomarkers. Circulating microRNAs and inflammation-related markers are promising candidates. Although miRNAs are implicated in cancer diagnostics, the role of miRNA-29a in ovarian cancer remains underexplored. Given that sST2 is elevated in several malignancies and is a direct target of miRNA-29a, concurrent evaluation may be informative. This pilot study compared serum miRNA-29a and sST2 levels in 23 ovarian cancer patients and 22 healthy female controls. miRNA-29a expression was quantified by real-time PCR (2^−ΔΔCt^), and sST2 was measured by ELISA; diagnostic performance was assessed using ROC analysis. miRNA-29a levels were significantly reduced (*p* < 0.05), whereas sST2 concentrations were significantly increased (*p* < 0.001) in patients versus controls. ROC analysis showed modest discrimination for miRNA-29a (AUC 0.678) and higher performance for sST2 (AUC 0.825). No significant correlation was observed between the two markers. These findings suggest that circulating miRNA-29a and sST2 may have biomarker potential in ovarian cancer; larger, well-designed studies are required to confirm clinical utility.

## 1. Introduction

Ovarian cancer is a malignant disease that represents one of the leading causes of cancer-related mortality among women, largely due to its frequent diagnosis at advanced stages [1,2]. The disease often progresses without noticeable symptoms, which significantly limits the possibility of early detection [3]. The most common form is high-grade serous carcinoma, which originates from the ovarian epithelium [4,5]. It occurs more frequently in the postmenopausal period, and several risk factors have been identified, including advanced age, obesity, late childbirth, postmenopausal hormone therapy, and a family history of ovarian, breast, or colorectal cancer [6,7]. Genetic mutations also play a significant role in the development of the disease [8,9]. It has been reported that individuals carrying a *BRCA1* (BReast CAncer gene 1) gene mutation have a significantly higher risk of developing ovarian cancer compared to those without the mutation [10].

Although CA-125 is widely used in clinical practice, its sensitivity is limited in early-stage disease and it may also be elevated in several benign gynecological conditions, reducing its diagnostic specificity [11,12,13]. Therefore, there is a continued need for complementary serum biomarkers that are minimally invasive, biologically informative, and stable in circulation. Changes in the expression levels of different miRNAs are considered potential new biomarker candidates for the diagnosis and treatment of diseases [14]. Circulating (miRNAs) have emerged as promising biomarker candidates due to their remarkable stability in serum and plasma, resistance to RNase degradation, and their ability to reflect disease-associated molecular alterations [15,16,17]. These properties make serum miRNAs attractive tools for cancer biomarker research.

MiRNAs are short, non-coding RNAs with a length of 18–22 nucleotides [18] that regulate mRNA expression through translational repression or mRNA degradation [19]. By participating in various biological processes such as proliferation, migration, and apoptosis, miRNAs exhibit different expression levels in different body regions, thereby regulating processes such as inflammation and cancer development [20,21]. Due to their ability to control gene expression, miRNAs are regarded as promising biomarkers for early diagnosis [22]. Furthermore, miRNAs have been shown to play diagnostic, prognostic, and therapeutic roles in ovarian cancer [23].

Although miRNA-29a has been extensively studied in various diseases such as epilepsy, scleroderma, and sepsis [24,25,26], its association with ovarian cancer has not yet been fully elucidated. There is still no consensus on whether miRNA-29a can reliably distinguish ovarian cancer from healthy tissue or other gynecological diseases. Most studies conducted to date have been performed on tumor tissues and cell lines obtained from ovarian cancer patients [27,28]. However, since serum samples can be obtained earlier and more easily compared to tumor tissues, the role of serum miRNAs in the early diagnosis of cancer has been gaining increasing attention [29]. Investigating the relationship between regulatory microRNAs such as microRNA-29a and tumor markers may contribute to identifying new pathways associated with cancer prognosis [30].

In epithelial ovarian cancers, low microRNA-100 expression has been shown to be associated with high CA-125 levels. This finding suggests that CA-125 may be regulated by microRNAs, which could be significant in terms of diagnosis or prognosis [31]. Therefore, in the present study, analyses were performed on serum samples to evaluate the relationship between miRNA-29a and ovarian cancer.

Additionally, the effects of different serum proteins on cancer development have long attracted attention due to their roles in elucidating disease prognosis. Interleukin-33 (IL-33) is defined as an “alarmin” released during cellular damage or stress, exerting its biological effects primarily through its specific receptor ST2 [32]. The ST2 receptor has two main isoforms: the membrane-bound ST2L (ST2) and the soluble form sST2. Binding of IL-33 to ST2L enhances cell survival signals, while sST2 binds to free IL-33, reducing its biological activity and thereby attenuating IL-33 signaling [33]. Disruptions in the IL-33/ST2L balance have been associated with pathologies such as chronic inflammation, tissue fibrosis, cancer development, and heart failure [34,35,36]. It has been reported that serum levels of sST2, which acts as a decoy molecule in the IL-33/ST2 pathway and represents another parameter of this study, vary among different cancer types [37]. In a cell line study, it was observed that suppression of the IL-33 gene in epithelial ovarian cancer (EOC) cells reduced their migratory and invasive potential, whereas full-length human IL-33 (fl-hIL-33) promoted the invasive, migratory, and proliferative capacities of EOC cells. This process was found to be inhibited by the IL-33 decoy receptor sST2 [38]. In another study related to sST2, tendinopathies were investigated, and sST2 was identified as a target of miRNA-29. It has been reported that, in tendon injuries, IL-33 release increases, which suppresses miRNA-29a and consequently elevates sST2 levels [39].

The mechanisms underlying these differences among studies have not been fully clarified. In this context, the present study investigated the serum levels of the sST2 protein in relation to the IL-33/ST2 axis, along with the expression levels of miRNA-29a in ovarian cancer and evaluated the possible association of these parameters with the disease. Assessing the diagnostic accuracy of miRNA-29a and sST2 may contribute to the development of diagnostic strategies. For this purpose, ROC (Receiver Operating Characteristic) curve analysis was planned. To the best of our knowledge, there are no studies in the literature that have evaluated the serum levels of miRNA-29a and sST2 in ovarian cancer using ROC analysis. ROC analysis is a commonly used method for evaluating the diagnostic accuracy of a test [40]. The area under the ROC curve (AUC) reflects the overall accuracy of the test and graphically represents the balance between sensitivity and specificity [41,42].

This pilot study aims to contribute to the existing knowledge gap by evaluating the serum levels and diagnostic performance of miRNA-29a in ovarian cancer using ROC analysis. Accordingly, serum levels of miRNA-29a and sST2 will be examined in ovarian cancer patients and healthy controls. We anticipate that the findings will support the biomarker potential of miRNA-29a and provide cautious additional evidence regarding the possible clinical relevance of sST2 in the context of ovarian cancer.

## 2. Materials and Methods

*Patient selection and data collection.* Individuals aged over 18 years were included in the study. The ovarian cancer group consisted of patients with a diagnosis confirmed clinically and/or histopathologically. The control group comprised healthy female volunteers aged over 18 years with no history of cancer or other serious illnesses; participants were randomly selected from the same hospital, and written informed consent was obtained prior to enrollment. Pregnant women, women with a history of other cancers, and those who had previously received chemotherapy were excluded from the study. The present study was approved by the Hitit University Non-Interventional Ethics Committee (approval no. 325/2024-25, date: 28 November 2024). The present study was conducted in accordance with the Declaration of Helsinki and signed informed consent forms were obtained from all participants.

*Data collection method.* Detailed demographic characteristics of all participants were documented. Venous blood samples were obtained from all participants using a standardized phlebotomy protocol. Within the scope of the study, peripheral blood was collected into plain vacuum gel tubes and centrifuged at 4500 rpm for 15 min in a refrigerated centrifuge to isolate serum. The resulting serum samples were aliquoted into sterile tubes and stored at −80 °C until miRNA analyses were performed.

*miRNA Isolation.* On the day of the experiment, serum samples were thawed and then clarified by centrifugation at 4500 rpm for 15 min at 4 °C. miRNA isolation was performed using the Nucleo Gene QuickEX Micro RNA Extraction Kit (LOT: NG20243711-NucleoGene, Istanbul, Türkiye) according to the manufacturer’s instructions. The purity and concentration of the isolated miRNAs were measured using the NanoDrop 2000 spectrophotometer (Thermo Fisher Scientific, Waltham, MA, USA).

*cDNA synthesis for reverse transcription (RT).* The transcription of miRNA samples into cDNA was performed using reverse transcriptase PCR (RT-PCR), following the instructions of the NucleoGene miRNA cDNA Synthesis Kit (LOT: NG20240577-Nucleo-Gene, Istanbul, Türkiye). RT-PCR was carried out using the MultiGene Optimax Thermal Cycler (cat. no. TC9610/TC9610-230; Labnet), New Jersey, USA. The quantity of synthesized cDNA was determined using the Qubit 3.0 Fluorometer (Thermo Fisher Scientific, Inc.) with the Qubit miRNA Analysis Kit. (cat. no. 32880-Thermo Fisher Scientific, Waltham, MA, USA) The samples were appropriately diluted with RNase-free water.

*Analysis of miRNA-29a Expression Levels.* The expression analysis of miRNA-29a in cDNA samples obtained from patient and control serum was conducted using the NucleoGene miRNA Gene Expression Assay (SYBR Green) Human mir29a (LOT: NG20240986-NucleoGene, Istanbul, Türkiye) with real-time PCR analysis on the ROTOR GENE device (QIAGEN, GmbH, Hilden, Germany). For the housekeeping control gene assay, the NucleoGene miRNA Gene Expression Assay U6 (SYBR Green) (LOT: NG20210896-NucleoGene, Istanbul, Türkiye) was used. The expression analysis of miRNA-29a and U6 was performed according to the instructions of the NucleoGene qPCR SYBR Green Master Mix (2X) kit (LOT: NG20240513-NucleoGene, Istanbul, Türkiye).

To determine the expression levels of miRNA-29a in the patient and control groups, ΔCt, ΔΔCt, and 2^−ΔΔCt^ values were calculated. The internal control (NucleoGene miRNA Gene Expression Assay U6 (SYBRGreen) was used to normalize ΔCt values and calculate the fold change in miRNA expression levels. The Livak formula (2^−ΔΔCt^) was applied to determine the miRNA levels. The ΔΔCt value was calculated by subtracting the mean ΔCt of the internal controls from the ΔCt of the target gene. Subsequently, the fold change was determined as 2^−ΔΔCt^ [43].

*ELISA Analysis.* Serum sST2 levels were measured using the Human sST2 ELISA Kit (ABT2802Hu, Human sST2, A.B.T. Ankara, Türkiye). This ELISA kit utilizes the Sandwich-ELISA method with a microplate pre-coated with a Human sST2-specific antibody. Samples were run in duplicates, and all procedures were performed according to the manufacturer’s instructions. Optical density was measured at 450 nm, and sST2 concentrations were determined using a standard curve. Absorbance values of standards, patient, and control samples were read using a SYNERGY/HTX Multi-Mode Reader (BioTek Instruments, Winooski, VT, USA). Sample concentrations (pg/mL) were calculated based on the standard curve.

*Statistical Analysis of Data.* Sample size was also calculated for the area under an ROC curve. It was assumed that the AUC of 0.76 for this particular test is significantly different from the null hypothesis value 0.5 (meaning no discriminating power), and α = 0.05 and power = 0.80, yielding at least n = 20 per group (total n = 40). The statistical analyses of the data obtained in the present study were conducted using the SAS v9.4 software package (SAS Institute, Inc., Cary, NC, USA). Quantitative variables are presented as median (range), whilst qualitative variables are presented as counts and percentages. The Kolmogorov-Smirnov and skewness values were used to assess the normal distribution of the data. Since the skewness coefficients for all variables were not between +3 and −3, it was concluded that the data did not show a normal distribution, meaning that non-parametric tests were utilized in the statistical analysis. The Mann–Whitney U test was applied for the comparisons between two independent groups.

To evaluate the effect size, Cohen’s d value was calculated. An effect size (Cohen d) value of less than 0.2 is defined as weak, 0.5 as medium, and greater than 0.8 as large. In the entire study, *p* < 0.05 was considered to indicate a statistically significant difference.

*(ROC) analysis.* The data were analyzed for sensitivity and specificity values obtained from ROC curves. To create optimal threshold values for the index tests, ROC curves were generated, and the AUC was determined. The ROC curve is a graph that plots the true positive rates (sensitivity) against the false positive rates (1-specificity) for different threshold values of the index tests. The closer the curve is to the top left corner of the graph, the higher the AUC and the greater the accuracy of the diagnostic test. In this study, ROC analyses were performed separately to determine the diagnostic accuracy of each biomarker. In the present study, the cutoff value was calculated from the AUC of 2^−ΔΔCt^ samples. The threshold value was selected using the Youden index (=Sensitivity + Specificity − 1). In the entire study, *p* < 0.05 was considered to indicate a statistically significant difference [44].

## 3. Results

The present study was conducted with a total of 45 cases. The average age of the patients was 50.00 ± 11.58 years, and the control group had an average age of 44.4 ± 9.28 years. The average age of the patient group was higher compared with that of the control group.

Clinical features of the patient group, such as family history, disease stage, menopausal status, and presence of metastasis, are detailed in Table 1.

**Figure 1:**
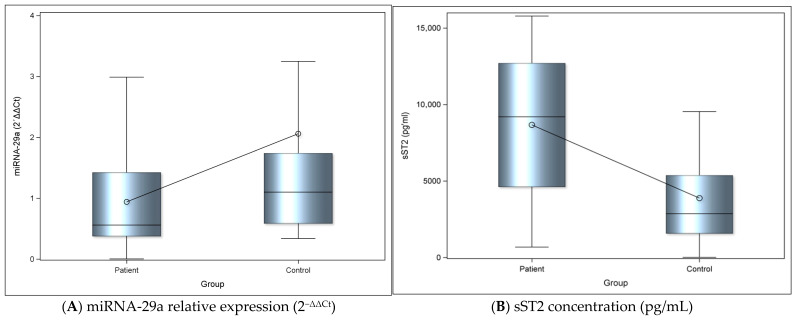
(**A**) The graph depicts the relative expression levels of miRNA-29a between groups. Bars represent mean values and error bars indicate standard deviation. Dots represent the averages. (**B**) The graph represents levels of sST2 between groups. Bars represent mean values, and error bars indicate standard deviation. Dots represent the mean of each group.

The relative expression levels of miRNA-29a were calculated using the 2^−ΔΔCt^ method in both patient and control groups. A statistically significant difference was observed between the 2^−ΔΔCt^ miRNA-29a expression levels in the patient and control groups, with the levels in the control group being significantly higher compared with those in the patient group (Cohen’s d = 0.58, indicating a moderate effect size) (Figure 1A; Table 2).

There was also a statistically significant difference between the sST2 levels in patient and control groups, with the levels in the control group being significantly lower compared with those in the patient group; the magnitude of this difference was large (Cohen’s d = 1.21), being significantly lower compared with those in the patient group (Figure 1B; Table 2).

**Figure 2:**
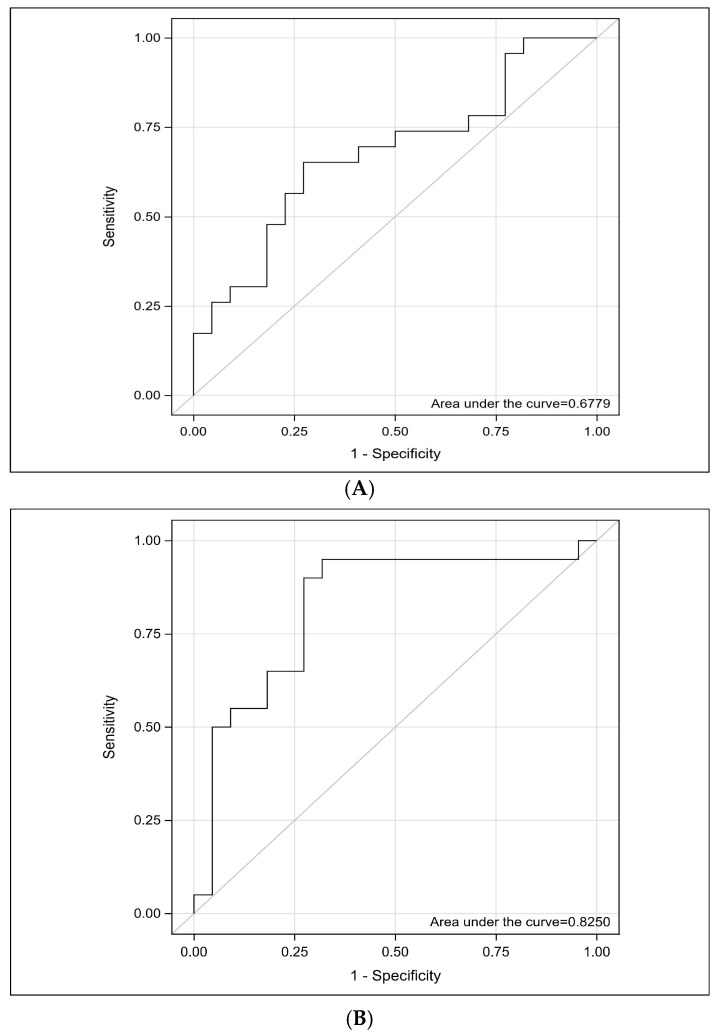
(**A**) ROC analysis of miRNA-29a. The ROC curve demonstrates the diagnostic performance of miRNA-29a in distinguishing patients with ovarian cancer from healthy controls. The analysis yielded an area under the curve of 0.6779 (95% CI, 0.5180–0.8377), indicating a moderate discriminative ability. The result was statistically significant with a *p* = 0.027. ROC; Receiver Operating Characteristic, miRNA, microRNA. (**B**) ROC analysis of sST2. The ROC curve demonstrates the diagnostic performance of sST2 in distinguishing patients with ovarian cancer from healthy controls. The analysis yielded an area under the curve of 0.8250 (95% CI, 0.6890–0.9610), indicating a moderate discriminative ability. The result was statistically significant with a *p* = 0.0001. ROC; Receiver Operating Characteristic, sST2; Soluble Suppression of Tumorigenicity 2.

The ability of the miRNA-29a biomarker to distinguish patients with ovarian cancer from healthy individuals was evaluated using the ROC curve and AUC value. The optimal threshold for 2^−ΔΔCt^, along with sensitivity and specificity, was calculated. An empirical ROC curve was generated using a non-parametric method in SAS software. The analysis yielded an AUC value of 0.678, with a 95% CI of 0.5180–0.8377 and a significance level of *p* = 0.027 (Table 3). These findings indicate that miRNA-29a 2^−ΔΔCt^ has a statistically significant discriminatory power in differentiating patients from healthy individuals (Figure 2A).

The ability of the sST2 biomarker to distinguish patients with ovarian cancer from healthy individuals was evaluated using the ROC curve and AUC value. The analysis yielded an AUC value of 0.8250, with a 95% CI of 0.6890–0.9610 and a significance level of *p* = 0.0001 (Table 3). These findings indicate that sST2 has a statistically significant discriminatory power in differentiating patients from healthy individuals (Figure 2B).

**Figure 3:**
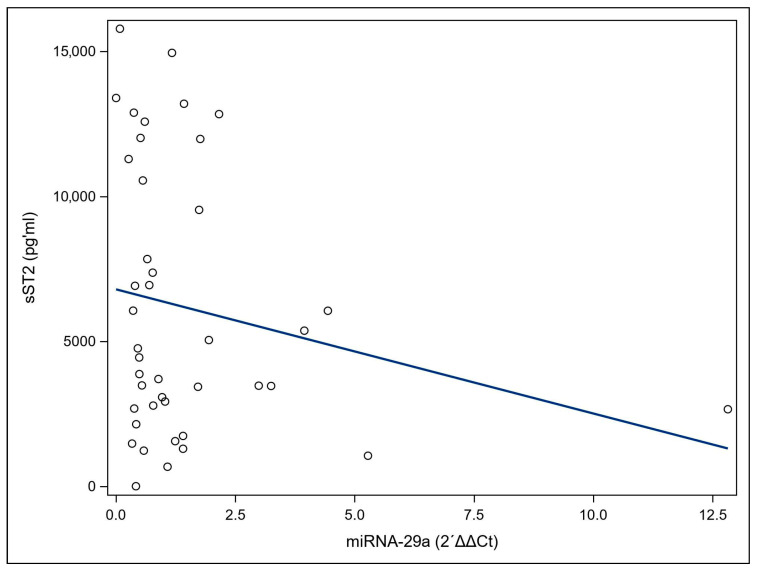
Spearman correlation of miRNA-29a and soluble suppression of tumorigenicity 2 (sST2) serum levels in ovarian cancer (OC) patients. The scatter plot matrix shows the correlations between pairs of the two markers, miRNA-29a, and sST2 as well as the corresponding Spearman coefficients (r) and *p*-values. (r = 0.2011, *p* = 0.2017).

A correlation analysis was performed to determine the relationship between miRNA-29a and sST2 levels in the patient group. There is no significant correlation between miRNA-29a and sST2 (Figure 3).

## 4. Discussion

Ovarian cancer, a heterogeneous disease with different histopathological types, is often difficult to detect early due to the fact that it typically presents clinical symptoms at advanced stages. However, early diagnosis is known to play a critical role in reducing high mortality rates [45,46]. With a five-year survival rate below 45%, ovarian cancer ranks eighth among cancer-related deaths in women [47]. Some genetic factors affect survival. BRCA1 and BRCA2 have been reported to be factors influencing survival in ovarian cancers [48]. When the biological underpinnings of ovarian cancer are examined, it becomes evident that molecular mechanisms play a crucial role in the initiation and progression of the disease. MicroRNAs exert significant regulatory effects on cell proliferation and tumor development; some function as oncogenic drivers, while others act as tumor suppressors. These characteristics underscore the potential of miRNAs as valuable diagnostic biomarkers and promising therapeutic targets [49,50,51].

In this context, the miRNA-29 family (miRNA-29a, miRNA-29b, and miRNA-29c) has emerged as an important regulatory group in cancer development. In particular, the proposition that miRNA-29a may serve as a potential therapeutic target and a promising biomarker in various tumor types [52] provided a compelling rationale for selecting this molecule as the focus of the present study. Moreover, the conspicuous scarcity of studies that comprehensively examine the association between miRNA-29a and ovarian cancer has further underscored the significance of investigating this molecule, providing an additional rationale for its selection in our study.

Current literature suggests that the miRNA-29 family may exert tumor-suppressive functions. Indeed, miRNA-29a has been reported to inhibit cellular proliferation and migration [53], and consistent with these findings, miR-29c-3p expression has been shown to be markedly reduced in ovarian cancer cases, with an even more pronounced decrease observed in recurrent tumors [54]. While these observations support the notion that the miRNA-29 family exhibits tumor-suppressive characteristics, some studies have also reported elevated miRNA-29a levels in certain (EOC) cases [55,56]. Such discrepancies may be attributed to methodological and biological variables, including sample type (tissue vs. serum), tumor stage, sample size, molecular subtypes, and intratumoral heterogeneity. As noted, the unique molecular architecture of different tissues may prevent a biological process occurring within the tissue from being directly reflected in serum measurements [57].

Moreover, miRNA-29a has been shown to suppress proliferation in cervical cancer cells [58], further supporting the possibility that its tumor-suppressive role is not limited to ovarian cancer but may extend to a broader range of gynecological malignancies. Although the number of studies assessing miRNA-29a expression in ovarian cancer remains limited, the decreasing trend observed in our study aligns with previous reports indicating that down-regulated miRNAs may play critical roles during the early stages of tumor development [52,53]. Taken together, these findings suggest that reduced miRNA-29a expression may be associated with tumor progression and aggressive biological behavior, indicating that miRNA-29a could function as an important regulatory element in the molecular pathology of gynecological cancers. However, considering that circulating miRNAs measured in serum may originate from multiple cellular sources and can be influenced by systemic host responses (e.g., inflammation), it would be appropriate to interpret the observed decrease in serum miRNA-29a levels with caution [59]. Further studies incorporating tissue-level validation may help to clarify the extent to which this finding reflects changes in tumor tissue expression.

In addition, when the biomarker sST2 is evaluated, evidence indicates an association between sST2 levels and thrombotic events in cancer patients, suggesting that sST2 may serve as an auxiliary biomarker in the diagnosis of venous thromboembolism [60]. Furthermore, sST2 expression has been reported to increase in response to specific characteristics of the tumor microenvironment [61]. Consistent with these observations, murine models have demonstrated that sST2 can promote orthotopic tumor growth [62]. Clinical studies also support these findings; for instance, significantly elevated sST2 levels have been observed in patients with gastric cancer, and this increase has been linked to metastatic processes [63]. Similarly, elevated sST2 concentrations have been reported in cases of hepatocellular carcinoma [64]. Moreover, activation of the IL-33/ST2 axis has been shown to correlate with poor survival outcomes in ovarian cancer patients [65].

As demonstrated by these studies and the available evidence, sST2 levels vary across different cancer types; however, the majority of recent findings indicate an overall trend toward increased expression of this molecule. Consistent with this pattern, our study also revealed that serum sST2 concentrations were significantly higher in ovarian cancer patients compared with the control group, suggesting that activation of the IL-33/ST2 axis may contribute to the pathogenesis of ovarian cancer.

To the best of our knowledge, no study has simultaneously or independently evaluated miRNA-29a and sST2 in the context of ovarian cancer. Examination of the literature investigating the relationship between the miRNA-29 family and sST2 indicates that type 2 cytokines regulate miR-29 expression, which in turn influences sST2 production. For instance, miRNA-29 molecules have been shown to suppress soluble ST2 secretion in bronchial epithelial cells [66]. Similarly, studies in tendinopathy models identified sST2 as one of the targets of miRNA-29; increased IL-33 release during tendon injury was reported to downregulate miRNA-29a expression, thereby leading to elevated sST2 levels [39,67].

Taken together, the present findings indicate that decreased miRNA-29a levels and increased sST2 concentrations may each be associated with ovarian cancer. However, the absence of a statistically significant correlation between the two markers may suggest that there is no relationship strong enough to support a shared or coordinated regulatory mechanism. This observation may be influenced by multiple factors, including disease heterogeneity and context-specific biological dynamics, the fact that serum measurements do not always reflect tissue activity, potential non-linear relationships, and limited statistical power. Therefore, these findings warrant validation in larger cohorts.

The observed decrease in miRNA-29a levels, accompanied by increased sST2 expression, further supports the notion that sST2 may contribute to tumor development and progression. The present findings indicate that circulating miR-29a and sST2 may have potential value as serum biomarkers in ovarian cancer. Importantly, this pilot study was not designed to investigate mechanistic interactions or causal relationships between these markers. The lack of correlation between miR-29a and sST2 suggests that they may provide independent or complementary diagnostic information, as has been reported for other biomarker panels [21,22].

In addition, the diagnostic performance of miRNA-29a and sST2 in distinguishing ovarian cancer patients from healthy controls was assessed using ROC curve analysis. The resulting AUC values were 0.6779 for miRNA-29a and 0.825 for sST2, indicating that both biomarkers possess moderate diagnostic power. Therefore, combining these biomarkers with other established diagnostic markers—such as Human Epididymis Protein 4 (HE4) or exosomal miRNAs—may enhance diagnostic accuracy and contribute to earlier detection of ovarian cancer [68].

One of the limitations of our study is that the mean age of the patient group was higher compared to the control group. In our study, age is considered a potential confounding factor between the patient and control groups, and this should be acknowledged as a limitation of this pilot study. Additionally, it is challenging to establish a completely healthy control group in this age range, as chronic conditions such as metabolic syndrome, cardiovascular diseases, or hormonal changes become more prevalent during the postmenopausal period [69,70]. Accordingly, the potential effect of age differences on miRNA-29a and sST2 levels is not thought to significantly influence the disease-specific biological changes of these biomarkers [71,72].

Secondly, although the sample size was determined using G*Power (3.1) analyses, the significant *p*-values obtained from the ROC analysis support the diagnostic potential of miRNA-29a and sST2 in ovarian cancer. However, the relatively small number of patients may partially limit the statistical power.

Finally, the relatively small number of early-stage (FIGO I–II) cases restricts the strength of conclusions regarding early detection and necessitates cautious interpretation. The inclusion of advanced-stage patients, however, allows an initial assessment of these biomarkers across a broader clinical spectrum and also raises the possibility that they may be explored in relation to disease monitoring or treatment response in future studies. Therefore, larger, stage-stratified cohorts are needed to improve generalizability and to better define the clinical utility of miRNA-29a and sST2 across disease stages.

A principal strength of this study is that, to our knowledge, it is the first to concurrently evaluate circulating miRNA-29a and serum sST2 in ovarian cancer. Assessing these markers in parallel provides an exploratory, hypothesis-generating framework for future studies to validate their diagnostic performance, either alone or in combination with established biomarkers.

The present findings suggest that circulating miR-29a and sST2 may have potential clinical value as serum biomarkers in ovarian cancer.

Although familial/genetic risk factors and tissue-level immunologic microenvironment were not directly assessed, this pilot study was specifically designed to evaluate miR-29a and sST2 simultaneously in ovarian cancer. Both miRNA-29a and sST2 are known to reflect immune regulation and inflammatory signaling at the systemic level, thereby providing indirect insight into disease-related immunologic processes without the need for invasive tissue-based analyses.

The absence of a correlation between miR-29a and sST2 may indicate that these markers reflect distinct biological processes and therefore could provide independent and/or complementary diagnostic information. Similar observations have been reported for other biomarker panels, where combining markers with non-overlapping profiles can improve diagnostic performance [73,74]. Accordingly, larger and preferably multi-center studies are warranted to validate the clinical utility of miR-29a and sST2, including their performance in combination with established markers such as CA-125.

## 5. Conclusions

Consequently, the concurrent evaluation of miRNA-29a and sST2 may contribute to a deeper understanding of ovarian cancer pathogenesis and facilitate the identification of novel biomarkers that could serve as a basis for diagnostic, prognostic, and therapeutic applications. Therefore, in the future, multicenter clinical studies with larger cohorts are needed, in which the expression levels of miRNA-29a and sST2 are analyzed together in both serum and tumor tissue.

## Figures and Tables

**Table 1:** Patients’ clinical features.

Patients	n, %
Family history of cancer	
Yes	9 (39.1%)
No	14 (60.9%)
Menopause	
Yes	16 (69.6%)
No	7 (30.4%)
Metastasis	
Yes	7 (30.4%)
No	16 (69.6%)
Stage	
I	6 (26.1%)
II	5 (21.7%)
III	8 (34.8%)
IV	4 (17.4%)

**Table 2:** Relative expression levels of miRNA-29a 2^−ΔΔCt^ and sST2 (pg/mL) levels.

Group	Patient (N = 23)	Control (N = 22)	*p*-Value
miRNA-29a 2^−ΔΔCt^ Mean (SD) Median (Range)			0.0410 *
	0.9 (0.92)	2.1 (2.78)	
	0.6 (0.03, 3.4)	1.1 (0.3, 12.8)	
sST2 (pg/mL) Mean (SD) Median			0.0003 *
	8669.0 (4421.20)	3880.3 (3412.20)	
	9198.3	2862.4	

* Range indicates the minimum and maximum values.

**Table 3:** ROC analysis of miRNA-29a (2^−ΔΔCt^) and sST2 (pg/mL).

	AUC	SE	95%CI		*p*	Cut Off	Sensitivity	Specificity
miRNA-29a	0.6779	0.0815	0.5180	0.8377	0.0270	0.6552	0.65217	0.72727
sST2	0.8250	0.0690	0.6890	0.9610	0.0001	3479.4	0.95	0.68182

## Data Availability

The raw data supporting the conclusions of this article will be made available by the authors on request.

## Abbreviations

The following abbreviations are used in this manuscript:

miRNA	Micro RNA
CA-125	Cancer Antigen 125
IL-33	Interleukin-33
ST2	Suppression of Tumorigenicity 2
sST2	Soluble ST2
EOC	Epithelial over cancer
ROC	Receiver Operating Characteristic
AUC	Area under curve
